# Supplementary figures and images for: Combining supervised and unsupervised analyses to quantify behavioral phenotypes and validate therapeutic efficacy in a triple transgenic mouse model of Alzheimer’s disease
Source: bioRxiv. 2024 Jun 8:2024.06.07.597924. Preprint. [Version 1] doi: 10.1101/2024.06.07.597924 (PMC11185760; doi:10.1101/2024.06.07.597924)

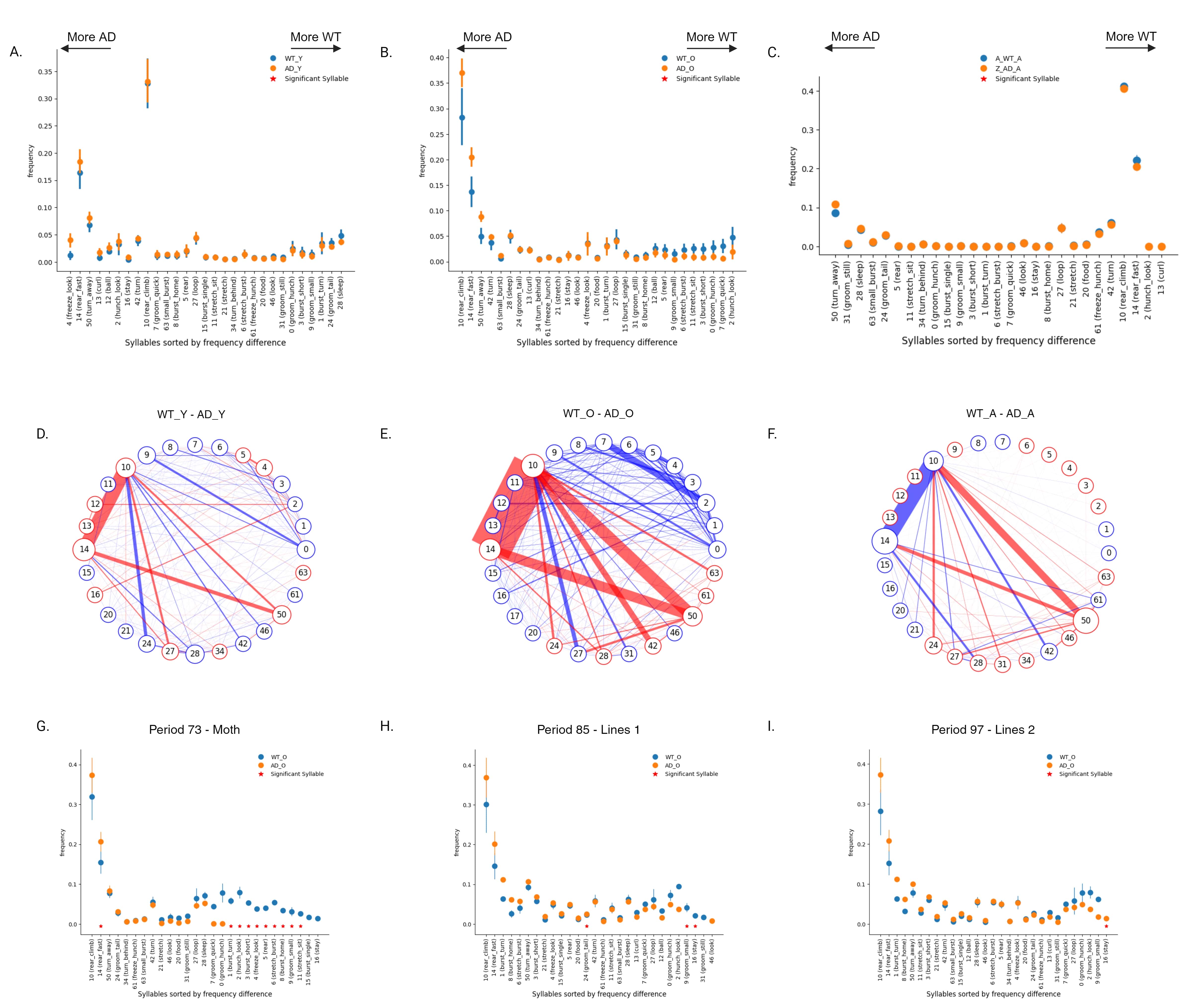

Supplement: Supplement 1 — Supplementary Figure 1. Analysis of syllables generated with Keypoint MoSeq. Frequency of syllables in A) young, B) old, and C) aged 3xTg-AD mice compared to WT mice. Syllable transition graphs of D) young, E) old, and C) aged 3xTg-AD mice compared to WT mice. Syllable frequencies of old mice during the first 10 minutes of the moth stimulus (G), the first set of moving lines in the home quadrant (H), and the second set of moving lines in the home quadrant (I). [file media-1.jpg]

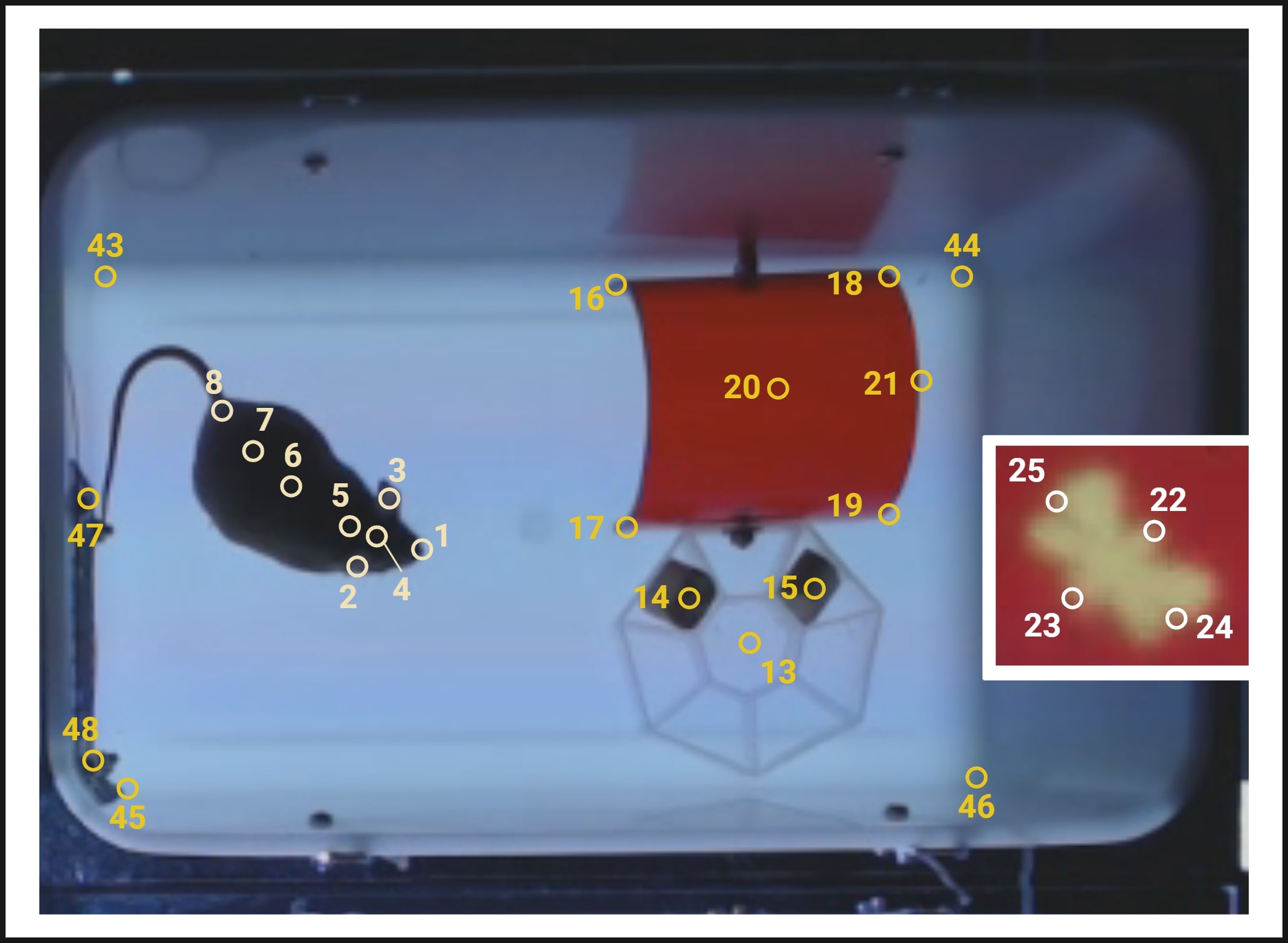

Supplement: Supplement 2 — Supplementary Figure 2. Mouse, cage, and stimuli markers used in the DeepLabCut model. 27 out of 48 markers were utilized for analysis in the current study. [file media-2.jpg]
