# Supplementary material for: Combining supervised and unsupervised analyses to quantify behavioral phenotypes and validate therapeutic efficacy in a triple transgenic mouse model of Alzheimer’s disease
Source: bioRxiv. 2024 Jun 8:2024.06.07.597924. Preprint. [Version 1] doi: 10.1101/2024.06.07.597924 (PMC11185760; doi:10.1101/2024.06.07.597924)
Supplement: 1 [file NIHPP2024.06.07.597924V1-supplement-1.pdf]

bioRxiv preprint doi: https://doi.org/10.1101/2024.06.07.597924; this version posted June 8, 2024. The copyright holder for this preprint (which was not certified by peer review) is the author/funder, who has granted bioRxiv a license to display the preprint in perpetuity. It is made available under aCC-BY 4.0 International license.

Supplementary Table 1. CatWalk gait analysis.

		Genotype		Age	
Measure	Type	Young	Old	WT	AD
Print length	LF	*↓	NS	NS	NS
	RF	*↓	NS	NS	NS
	LH	*↓	NS	NS	NS
	RH	NS	**↓	NS	NS
Toe spread	LH	NS	NS	NS	NS
	RH	NS	NS	NS	NS
Intermediate toe spread	LF	NS	NS	NS	NS
	RF	NS	NS	NS	NS
	LH	NS	NS	***↓	***↓
	RH	NS	NS	***↓	***↓
Print area	LF	*↑	NS	*↑	NS
	RF	NS	NS	*↑	NS
	LH	NS	NS	NS	NS
	RH	NS	NS	NS	NS
Maximum contact area	LF	**↑	NS	*↑	NS
	RF	NS	NS	*↑	NS
	LH	NS	NS	NS	NS
	RH	NS	NS	NS	NS
Mean intensity	LF	NS	***↑	NS	***↑
	RF	NS	***↑	*↑	***↑
	LH	NS	NS	NS	**↑
	RH	NS	NS	NS	***↑
Minimum intensity	LF	NS	***↑	NS	***↑
	RF	NS	***↑	NS	***↑
	LH	NS	NS	NS	**↑
	RH	NS	NS	NS	**↑
Stand	LF	NS	NS	NS	NS

bioRxiv preprint doi: https://doi.org/10.1101/2024.06.07.597924; this version posted June 8, 2024. The copyright holder for this preprint (which was not certified by peer review) is the author/funder, who has granted bioRxiv a license to display the preprint in perpetuity. It is made available under aCC-BY 4.0 International license.

	RF	NS	NS	NS	NS
	LH	NS	NS	NS	NS
	RH	NS	NS	NS	*↑
Paw angle body axis	LF	**↑	NS	NS	NS
	RF	*↑	NS	NS	NS
	LH	NS	*↓	NS	*↓
	RH	NS	NS	NS	NS
Step cycle	LF	NS	NS	NS	NS
	RF	NS	NS	NS	NS
	LH	NS	NS	NS	NS
	RH	NS	NS	NS	*↑
Support	Lateral	NS	NS	NS	NS
	Girdle	NS	NS	NS	NS
	Diagonal	NS	NS	NS	NS
	Four	NS	NS	NS	NS
	Three	NS	NS	NS	NS
	Single	NS	NS	NS	NS
	Zero	NS	NS	NS	NS
Base of support -front paws		NS	NS	NS	NS
Base of support -hind paws		NS	NS	NS	*↑
Duration		NS	NS	NS	NS
Cadence		NS	NS	NS	NS
Number of steps		*↓	NS	**↓	NS
Step sequence		*↓	NS	***↓	NS

↑ and ↓ denote an increase or decrease in 3xTg-AD mice compared to WT mice (Genotype), or an increase or decrease in old mice compared to young mice (Age), respectively.
